# Changes in a Cone Opsin Repertoire Affect Color-Dependent Social Behavior in Medaka but Not Behavioral Photosensitivity

**DOI:** 10.3389/fgene.2020.00801

**Published:** 2020-08-12

**Authors:** Nodoka Kanazawa, Mayuko Goto, Yumi Harada, Chiaki Takimoto, Yuuka Sasaki, Tamaki Uchikawa, Yasuhiro Kamei, Megumi Matsuo, Shoji Fukamachi

**Affiliations:** ^1^Department of Chemical and Biological Sciences, Japan Women’s University, Bunkyō, Japan; ^2^National Institute for Basic Biology, Okazaki, Japan; ^3^School of Life Sciences, The Graduate University for Advanced Studies (SOKENDAI), Okazaki, Japan

**Keywords:** medaka (*Oryzias lapites*), reverse genetics, color discrimination, spectral sensitivity, sensory drive, short wavelength sensitive gene

## Abstract

Common ancestors of vertebrates had four types of cone opsins: short-wavelength sensitive 1 (SWS1), SWS2, rhodopsin 2 (RH2), and long-wavelength sensitive (LWS) types. Whereas fish and birds retain all the types, mammals have lost two of them (SWS2 and RH2) possibly because of their nocturnal lifestyle during the Mesozoic Era. Considering that the loss of cone opsin types causes so-called color blindness in humans (e.g., protanopia), the ability to discriminate color by trichromatic humans could be lower than that in potentially tetrachromatic birds and fish. Behavioral studies using color-blind (*cone opsin*-knockout) animals would be helpful to address such questions, but it is only recently that the genome-editing technologies have opened up this pathway. Using medaka as a model, we introduced frameshift mutations in *SWS2* (*SWS2a* and/or *SWS2b*) after detailed characterization of the loci *in silico*, which unveiled the existence of a GC–AG intron and non-optic expressed-sequence-tags (ESTs) that include *SWS2a* in part. Transcripts from the mutated *SWS2* loci are commonly reduced, suggesting that the *SWS2a/b*-double mutants could produce, if any, severely truncated (likely dysfunctional) SWS2s in small amounts. The mutants exhibited weakened body color preferences during mate choice. However, the optomotor response (OMR) test under monochromatic light revealed that the mutants had no defect in spectral sensitivity, even at the absorbance maxima (λ_max_) of SWS2s. Evolutionary diversification of cone opsins has often been discussed in relation to adaptation to dominating light in habitats (i.e., changes in the repertoire or λ_max_ are for increasing sensitivity to the dominating light). However, the present results seem to provide empirical evidence showing that acquiring or losing a type of cone opsin (or changes in λ_max_) need not substantially affect photopic or mesopic sensitivity. Other points of view, such as color discrimination of species-specific mates/preys/predators against habitat-specific backgrounds, may be necessary to understand why cone opsin repertories are so various among animals.

## Introduction

Colors are virtual images evoked in the brain by light spectra received at the retina. Physically different spectra (e.g., yellow monochromatic light and red/green dichromatic light) make a person evoke an identical color, whereas an identical spectrum makes different people (e.g., monochromats, dichromats, and trichromats) evoke different colors. Animals possess various sets of photoreceptors in the retina. Thus, different animals may evoke different colors when looking at the same object, i.e., the world could differently be colored for different species or even individuals.

The mechanism for color perception in humans has been studied extensively (Solomon and Lennie, 2007; Conway, 2009; Neitz and Neitz, 2011). Under daylight, light is received by three types (or more precisely, two types with one subtype; see below) of visual pigments (cone opsins) and converted to an electronic signal of three channels (Young, 1802). This signal is then converted to a two-dimensional value defined by the red–green and blue–yellow axes (Hering, 1920) *via* complex neural networks of horizontal, bipolar, amacrine, and ganglion cells (Thoreson and Dacey, 2019), which is sent to the visual cortex of the brain where colors are evoked. However, this mechanism (still not fully understood, particularly the processing in the brain) will explain color perception only in a part of the Old World monkeys (Catarrhini). These animals, as do other mammalian species (except for monotremes), possess two types of cone opsins: short-wavelength sensitive 1 (SWS1) and long-wavelength sensitive (LWS) types (Bowmaker, 1998), which are often referred to as (ultra)violet and red opsins, respectively. Common ancestors of the catarrhine monkeys duplicated the *LWS* gene, accumulated missense substitutions, diversified absorbance maxima (λ_max_) of the proteins, and acquired so-called trichromacy. The cone cells in their retina are classified morphologically into two types (expressing SWS1 or either of the LWS subtypes), which are arranged largely at random (Viets et al., 2016).

Fish (and birds) possess two additional types of cone opsins, which mammals have lost during their nocturnal lifestyle in the Mesozoic Era: SWS2 and rhodopsin 2 (RH2), which are often referred to as blue and green opsins, respectively. These four types of cone opsins are expressed in four types of cone cells, which are regularly arranged in the retina (i.e., retinal mosaics; Tohya et al., 2003; Carleton et al., 2020). Although spatiotemporal patterns of cone opsin expression in fish are complex (Tsujimura et al., 2007; Dalton et al., 2014; Zimmermann et al., 2018), the retinal mosaic *per se* should suggest that the four types of cones could function coordinately for tetrachromacy, which has been suggested or actually demonstrated in some species (see Thoreson and Dacey, 2019 and references therein). Various attempts have been made to understand the vision of fish (e.g., spectral absorbance of opsin molecules, microspectrophotometry, electroretinography, theoretical modeling, and behavioral assays), but we still do not understand the mechanism or how differently their worlds would be colored in comparison with ours.

Recent genome-editing technologies have made it possible to establish color-blind animals by knocking out the *cone opsin* genes. Considering that color-blind patients have made great contributions to our understanding of the human trichromacy (Neitz et al., 1999; Neitz and Neitz, 2014), color-blind animals should provide promising opportunities for dissecting characteristics and mechanisms for color perception in animals. However, such resources are scarce at present. In mice, an *SWS1*-knockout strain is available (Greenwald et al., 2014), but dichromatic mice would not fit as a model for studying tetrachromacy. In zebrafish, there is a strain that specifically lacks the red cones, but the mutation is not in the *cone opsin* gene and the mutants are lethal at the larval stages (Taylor et al., 2005).

We recently established medaka strains that lack LWS and made some interesting findings by analyzing the mutants using a monochromatic light source, the Okazaki Large Spectrograph (OLS; Watanabe et al., 1982). For example, (i) whereas many previous studies at the molecular, cellular, physiological, behavioral, or theoretical levels ignored retinal inputs at λ > 700 nm, the wild-type medaka (as do some other fish species: M.M., Y.K., and S.F., in preparation) fully exhibit the optomotor response (OMR) at λ > 800 nm; (ii) the light-adapted, but not dark-adapted, *lws* mutants (*LWSa/b*-double knockouts) significantly reduced the OMR at λ ≥ 740 nm, demonstrating that the rod-dependent scotopic vision is dysfunctional in light-adapted medaka, and that not only LWSs (λ_max_ at 561–562 nm) but also RH2s (λ_max_ at 452–516 nm) can absorb and make medaka respond behaviorally to light at wavelengths > 200 nm longer than the λ_max_; (iii) an in-frame fusion of the tandemly located *LWSa* and *LWSb* loci, which are nearly identical in nucleotide sequence because of a recent gene conversion (i.e., a decreased copy number of the *LWS* genes), did not at all reduce red-light sensitivity, obscuring the necessity of their coexistence and coexpression; and (iv) a premating sexual isolation between body color variants, the *color interfere* (*ci*) mutant that lacks somatolactin alpha (SLα) and the actin beta (Actb)-SLα:green fluorescent protein (GFP) transgenic fish that overexpresses SLα, which can be observed convincingly under white, but not monochromatic lights (with half bandwidth of ± 5 nm), was significantly relaxed in the *lws* mutants under white light, suggesting the importance of LWSs in the color-dependent (not luminance-dependent) mate choice (Fukamachi et al., 2009a; Utagawa et al., 2016; Homma et al., 2017; Ikawa et al., 2017; Kamijo et al., 2018; Matsuo et al., 2018; Harada et al., 2019).

These kinds of reverse-genetic studies can provide direct empirical evidence for a causal relationship between cone opsin repertoire and animal behavior. Comparative studies using different species or heterospecific populations with different cone opsin repertoires could be another choice, but effects from other polymorphic genes (i.e., misidentification of a causal relationship) cannot be excluded. In this regard, the invention of genome-editing technologies provides a newly discovered and powerful strategy for assessing whether or not and to which degree each member of a cone opsin repertoire actually contributes to visual-dependent behaviors of animals.

In the present study, we focused on SWS2, whose ecological/evolutionary importance has been discussed in fish (Cortesi et al., 2015; Marques et al., 2017). Unlike LWSa and LWSb, the identity between SWS2a and SWS2b drops to 77.0% (271/352 amino acids) and their λ_max_ is distinctly different (439 and 405 nm, respectively) (Matsumoto et al., 2006). Therefore, we established *SWS2a*-single, *SWS2b*-single, and *SWS2a/b*-double knockout medaka and assessed each phenotype using the methods we previously developed for assessing the *lws* mutants (Homma et al., 2017; Kamijo et al., 2018; Matsuo et al., 2018; Harada et al., 2019). Results of the mate-choice experiment and the OMR test will respectively tell us whether or not the *sws2* mutants have defects (i.e., SWS2s play essential roles) in color discrimination and photosensitivity.

## Results

### The Medaka *SWS2* Loci

**FIGURE 1:**
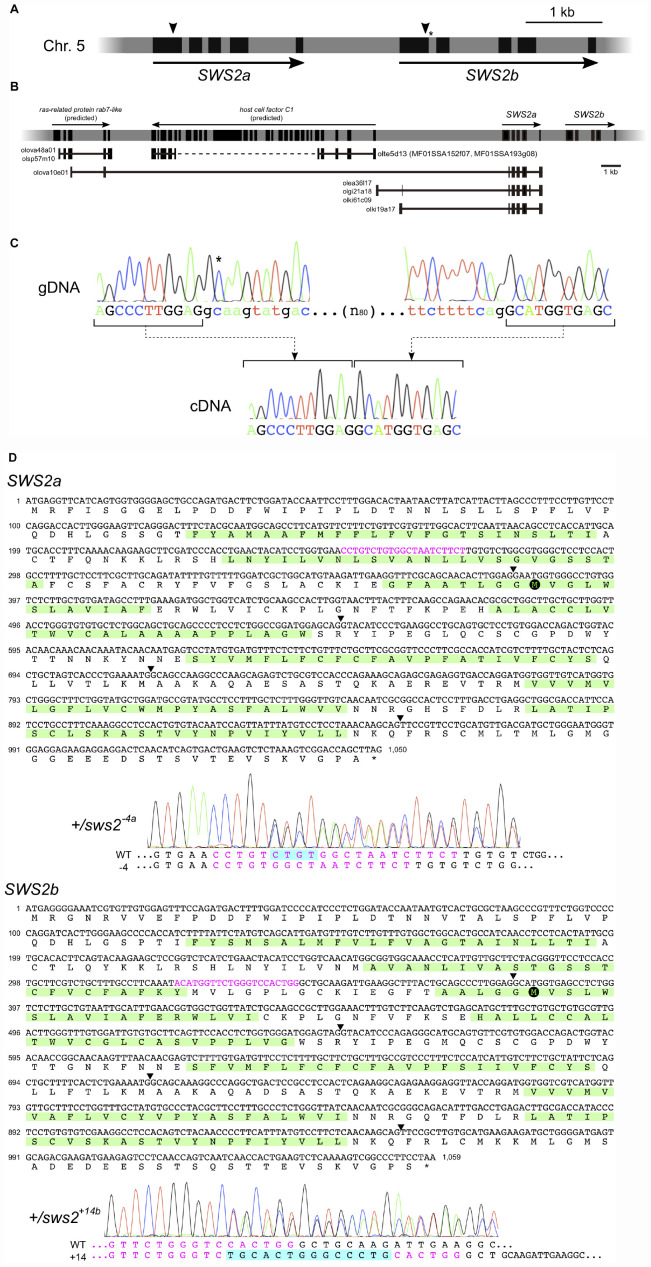
The medaka *short-wavelength sensitive (SWS)2a* and *SWS2b* loci. **(A)** A diagram of the *SWS2* loci on the chromosome 5. The paralogs are concatenated tandemly in a head-to-tail manner. Black boxes represent coding regions (from the start codon to the stop codon). Arrowheads are approximate positions of target sequences for CRISPR/Cas9. An asterisk shows a position of the splice donor of the GC–AG intron (the first intron of *SWS2b*). **(B)** The expressed-sequence-tag (EST) clones containing a part of *SWS2a*. A horizontal gray bar with black boxes represents a part of chromosome 5. Black boxes indicate transcribed regions including untranslated regions (UTRs) (those of *SWS2*s do not include UTRs as **A**). Horizontal black lines with black boxes are ESTs found in the database at the National Bioresource Project (NBRP) medaka. *ras-related protein rab7-like* (XM_020703466 and XM_020703467) and *host cell factor C1* (XM_011475416) have been registered as predicted genes in GenBank, and corresponding ESTs (olova48a01, olsp57m10, olte5d13, MF01SSA152f07, and MF01SSA193g08) are found presently existing in the database. A dotted line is the region not sequenced in the ESTs. A total of five EST clones (olova10e01, olea36l17, olgi21a18, olki61c09, and olki19a17) contained a part of *SWS2a*. Three of the five clones (olea36l17, olgi21a18, and olki61c09) additionally contain upstream intergenic regions and a part of the fourth intron. olki19a17 possesses a different intergenic region, and olova10e01 has a part of exons of the upstream *ras-related protein rab7-like* gene. **(C)** The GC–AG intron. Electropherograms of the genomic (top) and complementary (bottom) DNA sequences are shown. Uppercase indicates the first and second exons of *SWS2b*, and lowercase indicates the first intron. The C residue with an asterisk does not follow the GT–AG rule, but the intron is spliced out. **(D)** mRNA sequences of *SWS2a* and *SWS2b* and the *sws2* mutations. Translated amino acid sequences are also shown, with the transmembrane domains (predicted by TMHMM; http://www.cbs.dtu.dk/services/TMHMM/) highlighted by green. Arrowheads are positions of introns. Target sequences are shown in magenta, and downstream methionines (potential initiation sites of translation) are highlighted by black circles. Electropherograms obtained for the *sws2^– 4a^* and *sws2*^+14b^ heterozygotes are shown. Note that peaks are doubled from the position where the frameshift mutation is introduced. The sequences of mutated alleles (bottom) are determined by subtracting the known sequences of the wild type (top) from the doubled electropherogram.

Before the genome editing, we examined genomic sequences of the medaka *SWS2a* and *SWS2b* loci (Figure 1A) that are available at the GenBank (AB223056 and AB223057, respectively) and the UTGB (version 2.2.4)^1^ databases. The GenBank sequences (2,324 bp of *SWS2a* and 3,404 bp of *SWS2b*) were of the HNI strain, and the *SWS2a* sequence was identical to the whole-genome sequence of HNI in the UTGB, except that the fourth intron (249 bp) seemed to be of the Hd-rR strain for some unknown reason. We detected a total of six mismatches [five substitutions and one insertion/deletion (ins/del)] in *SWS2b*, one of which is located at the splice-donor site of the first intron (Figure 1A), i.e., GT in the GenBank, but GC in the UTGB.

This substitution seemed not to be negligible because it may cause mis-splicing. Hence, we screened the expressed-sequence-tag (EST) database provided by the National Bioresource Project (NBRP) Medaka^2^ to confirm the exon–intron boundaries. However, no EST (of 730,259 entries) was identical to *SWS2b*. This database search identified some interesting clones containing a part of the first and the entire second to fifth exons of *SWS2a*, which are connected to an upstream gene or intergenic regions (Figure 1B). Whether these strange ESTs were artifacts during library construction or indeed expressed and function in the organs from which they were isolated (i.e., the kidney, ovary, or gill) remains unknown.

Given that the examinations *in silico*, as described above, do not confirm the open-reading frames (ORFs) of *SWS2a* or *SWS2b*, we experimentally determined the ORFs using the *ci* strain, whose genome was going to be edited in this study. Direct sequencing of RT–PCR products revealed that the ORFs of *SWS2a* and *SWS2b* consist of 1,047 and 1,056 bp (excluding the stop codon), respectively, as reported for HNI in the GenBank database. These ORFs are split into five exons with conserved exon–intron boundaries, meaning that the first intron of *SWS2b* is indeed spliced out. Then, we examined the splice donor of the first intron by direct sequencing of genomic PCR products and found that GC is correct, as reported in the UTGB database (Figure 1C). Hence, although the first intron of *SWS2b* does not follow the GT–AG rule, it functions as an GC–AG intron, which has been reported from various species, including humans (Thanaraj, 2001).

### Frameshift Mutations on the *SWS2* Genes

The unusual ESTs in Figure 1B suggest that a frameshift mutation for knocking out *SWS2a* needs to be induced on the first exon (otherwise, these potentially functional ESTs could also be knocked out). We designed gRNAs that target either *SWS2a* or *SWS2b* [their ORFs were 75.1% (793/1,056) identical] for the CRISPR/Cas9 system and microinjected either or both of the gRNAs with the *Cas9* mRNA into fertilized eggs of the *ci* and Actb-SLα:GFP strains.

Among a total of 29 G_0_ adults that successfully passed ins/del mutations to their F_1_s, we used eight G_0_s to obtain a total of 77 F_1_s, 15, 11, and 13 of which were heterozygous for the *SWS2a*-single, *SWS2b*-single, and *SWS2a/b*-double mutations, respectively (Tables 1–3). These ins/del mutations were classified into 19 haplotypes, four, four, and five of which were *SWS2a*-single, *SWS2b*-single, and *SWS2a/b*-double frameshift mutations, respectively. The F_1_ fish with the frameshift mutation/s were basically backcrossed with *ci* or Actb-SLα:GFP because F_1_s possessing an identical haplotype were scarce and insufficient for intercrossing (e.g., only one F_1_ for *sws2^+29a^*, three F_1_s for *sws2^+14b^*, but only male fish, etc.; see Tables 1–3). These backcrosses should basically reduce the risk of off targets.

**TABLE 1:** Single ins/del mutations on *SWS2a* inherited from G_0_ to F_1_.

Allele	Sequence*	No. of F_1_
wt	AACCTGTCTGTGGCTAATCTTCTTGTGTCTG	–
–8a	AACCTGTC——–ATCTTCTTGTGTCTG	1
–4a	AACCTGT—-GGCTAATCTTCTTGTGTCTG	9
+4a	AACCTGTCaacctgtgaCTAATCTTCTTGTGTCTG	2
+15a	AACCTGTCTaatctgtctaataaccTGGCTAATCTTCTTGTGTCTG	2
+29a	AACtgaatcctggtgaacctggtgaacctggtgaacCTGTGGCTAATCTTCTTGTGTCTG	1
	Total	15

*Inserted or deleted nucleotides are shown by lower cases or hyphens, respectively. The target sequence is underlined. del, deletion; ins, insertion; SWS, short-wavelength sensitive; wt, wild type.

**TABLE 2:** Single ins/del mutations on *SWS2b* inherited from G_0_ to F_1_.

Allele	Sequence*	No. of F_1_
wt	ACATGGTTCTGGGTCCACTGGGCTGCAAGATTGAAGGC TTTAC	–
–16b	ACATGGTTCTGGGTCtgcactgggc—————-AC	1
–9b	ACATGGTTCTGGG———CTGCAAGATTGAAGGCTTTAC	1
–9b (2)	ACATGGTTCTGGGT———TGCAAGATTGAAGGCTTTAC	3
–8b	ACATGGTTCTGGGTC——–TGCAAGATTGAAGGCTTTAC	2
–2b	ACATGGTTCTGGGTt–CTGGGCTGCAAGATTGAAGGCTTTAC	1
+14b	ACATGGTTCTGGGTCtgcactgggcactgCACTGGGCTGCAAGATTGAAGGCTTTACTG	3
	Total	11

*Inserted or deleted nucleotides are shown by lower cases or hyphens, respectively. The target sequence is underlined. del, deletion; ins, insertion; SWS, short-wavelength sensitive; wt, wild type.

**TABLE 3:** Double ins/del mutations on *SWS2a* and *SWS2b* inherited from G_0_ to F_1_.

Haplotype	Sequence*	No. of F_1_
wt	*a*: AACCTGTCTGTGGCTAATCTTCTTGTGTCTG	−
	*b*: ACATGGTTCTGGGTCCACTGGGCTGCAAGATTGAAGGCTTTAC	
–6a–4b	*a*: AACCTG——GCTAATCTTCTTGTGTCTG	2
	*b*: ACATGGTTCTGGG—-CTGGGCTGCAAGATTGAAGGCTTTAC	
–4a–103b	*a*: see Table 1	1
	*b*: ACATGGTTCTGGGTCCACTGGGCTGCAAGA————-**	
–4a–9b	*a*: see Table 1	2
	*b*: see Table 2	
–4a–2b	*a*: see Table 1	1
	*b*: see Table 2	
–4a+2b	*a*: see Table 1	1
	*b*: ACATGGTTCTGGGTacaCACTGGGCTGCAAGATTGAAGGCTTTAC	
–4a+5b	*a*: see Table 1	2
	*b*: ACATGGTTCTGGGctgcaagatCTGGGCTGCAAGATTGAAGGCTTTAC	
+1a+14b	*a*: AACCTGTCTtGTGGCTAATCTTCTTGTGTCTGG	1
	*b*: see Table 2	
+4a–3b	*a*: AACCTGTCTaaacCTGGCTAATCTTCTTGTGTCTG	3
	*b*: ACATGGTTCTtg—CACTGGGCTGCAAGATTGAAGGCTTTAC	
	Total	13

*Inserted or deleted nucleotides are shown by lower cases or hyphens, respectively. The target sequences are underlined. **Detailed sequence not shown. This relatively large deletion was detected by not HMA but longer PCR for direct sequencing. del, deletion; HMA, heteroduplex mobility assay; ins, insertion; SWS, short-wavelength sensitive; wt, wild type.

Considering positions of the CRISPR/Cas9 target sequences and downstream codons for methionine (i.e., potential sites for translational initiation), SWS2 proteins translated from the frameshifted mRNA must severely be truncated losing multiple transmembrane domains, which should likely be dysfunctional as a G protein-coupled receptor (Figure 1D).

### The *sws2* Mutants

When the heterozygous F_1_s are intercrossed, an expected genotype ratio among their F_2_ siblings is *SWS2*^+/+^:*SWS2^+/–^*:*SWS2^–/–^* = 1:2:1, which was indeed observed for all the eight haplotypes (three *SWS2a*-single, two *SWS2b*-single, and three *SWS2a/b*-double frameshift mutations; Table 4). For example, we obtained a total of 33 adults by intercrossing the *sws2^–4a^* (a four-base deletion on *SWS2a*) heterozygotes and the genotype ratio was 11:12:10, which was not significantly different from the expected 8:17:8 (*P* = 0.284, chi-square test). Although the number of offspring per family (i.e., 11–67 F_2_s) was not always sufficiently large enough for statistical analysis, the overall ratio became 69:134:65, which is very close to the expected 67:134:67 (*P* = 0.942). Body colors of the *sws2*-mutant siblings are not distinguishable from those of the wild-type or heterozygous siblings on either *ci* or Actb-SLα:GFP background, at least for humans.

**TABLE 4:** Genotypes of adult littermates obtained by crossing *SWS2*^+/–^ heterozygotes.

Allele/haplotype	# of fish with genotype of			P*
	
	*SWS2*^+/+^	*SWS2*^+/–^	*SWS2^–/–^*	
–4a	11	12	10	0.284
+8a	20	34	13	0.478
+29a	3	8	3	0.867
–8b	3	6	2	0.873
+14b	9	27	15	0.452
–4a–2b	5	18	7	0.480
–4a+2b	5	13	9	0.543
–4a+5b	11	17	14	0.377
Total	69	134	65	0.942

*Chi-square tests with the null hypothesis that “SWS2^+/+^:SWS2^+/–^:SWS2^–/–^ = 1:2:1.” SWS, short-wavelength sensitive.

Besides the eight haplotypes, we crossed female homozygotes for the *sws2^+1a+14b^* mutation (one-base and 14-base insertions on *SWS2a* and *SWS2b*, respectively) with heterozygous male fish. A genotype ratio of their siblings at adult stages was *SWS2^+/–^*:*SWS2^–/–^* = 7:6, which is not significantly different from the expected 6.5:6.5 (*P* = 0.782).

These results demonstrate that the lack of either or both SWS2s does not affect the viability of medaka, at least under laboratory conditions. All the nine lines are available as frozen sperm at the NBRP Medaka as the following names and IDs: *sws2^–4a^* (MT1158), *sws2^+8a^* (MT1159), *sws2^+29a^* (MT1160), *sws2^–8b^* (MT1161), *sws2^+14b^* (MT1162), *sws2^–4a–2b^* (MT1163), *sws2^+1a+14b^* (MT1178), *sws2^–4a+5b^* (MT1179), and *sws2^–4a+2b^* (MT1180).

### *Cone Opsin* Expressions in the *sws2* Mutants

**FIGURE 2:**
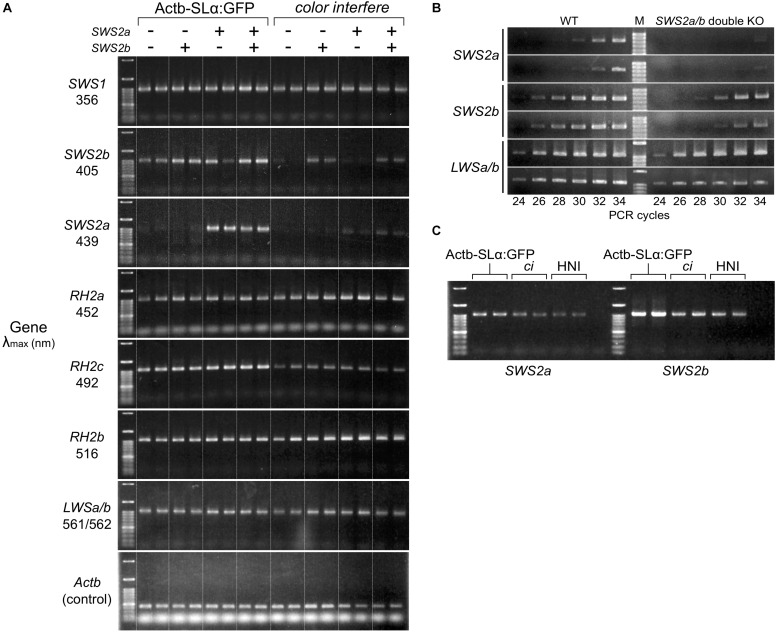
Expression of the *cone opsin* genes in the *short-wavelength sensitive* (*sws*)*2* mutants. **(A)** RT-PCR of all the *cone opsin* genes in medaka. Gene names and λ_max_ of the proteins are shown on the left. *Long-wavelength sensitive* (*LWS*)*a* and *LWSb* could not be analyzed separately because of their high sequence identity. Genomic backgrounds [actin beta (Actb)-somatolactin alpha (SLα):green fluorescent protein (GFP) or *color interfere* (*ci*)] and genotypes for the *SWS2* loci [ + (homozygotes of the wild-type allele) or – (homozygotes of the mutated allele)] are shown on the top. From the left lane in each genomic background, results of the *sws2^– 4a+2b^*, *sws2^– 4a– 2b^*, *sws2*^+29a^, *sws2*^+8a^, *sws2^– 8b^*, *sws2*^+14b^, and two wild-type fish are shown. Note that expression is largely the same in the 16 individuals in terms of *SWS1*, *rhodopsin (RH)2a*, *RH2b*, and *LWSa/b* (that of *RH2a* may fluctuate slightly). Apparent differences are found in the expression of *SWS2b*, *SWS2a*, and *RH2c*. In *RH2c*, the expression is commonly higher in Actb-SLα:GFP than the expression in *ci*. In *SWS2b* and *SWS2a*, the expression is also higher in Actb-SLα:GFP than it is in *ci* (see results in the wild type). Additionally, the *SWS2* expression differs depending on the genotype, i.e., when mutated, the expression is decreased on both genomic backgrounds. The number of PCR cycles was 30 for *SWS2*s, 26 for *SWS1*, *RH2*s, and *LWS*s, and 20 for *Actb*. **(B)** Examples of the stepwise RT-PCR for determining the appropriate number of PCR cycles (i.e., before plateau) for **(A)**. Here, results at every two cycles between 24 and 34 cycles of two wild type and two *SWS2a/b*-double mutants (*sws2^– 4a+2b^* and *sws2^– 4a– 2b^*) with the *ci* background are shown for three genes (*SWS2a*, *SWS2b*, and *LWSa/b*). Note delayed amplifications in the *sws2* mutants for *SWS2a* and *SWS2b*, but not *LWSa/b*. We used forward primers different from those listed in Table 6 (namely, f: 5′-AACAAGAAGCTTCGATCCCA for *SWS2a* and f: 5′-TTGTTGCTTCTACGGGTTCC for *SWS2b*), which is why products shorter than those in **(A)** were amplified. **(C)** RT-PCR of the *SWS2* genes in three wild-type (i.e., with no mutations on any *cone opsin* genes) strains that express SLα differently. The Actb-SLα:GFP, *ci*, and HNI strains excessively, never, and ordinarily express SLα, respectively (Fukamachi et al., 2004, 2009b). The expression of *SWS2a* and *SWS2b* is stronger in Actb-SLα:GFP than in *ci* as in **(A)**. Expression in HNI seems to be more similar to that in *ci* than to that in Actb-SLα:GFP, suggesting that the expression of *SWS2* is enhanced in Actb-SLα:GFP (rather than suppressed in *ci*; see section “Discussion”).

Using two *SWS2a*-single (*sws2^+8a^* and *sws2^+29a^*), two *SWS2b*-single (*sws2^–8b^* and *sws2^+14b^*), and two *SWS2a/b*-double mutants (*sws2^–4a–2b^* and *sws2^–4a+2b^*) as representatives (*n* = 1 each), we compared the expression of full-length (more precisely, all-coding exon-containing) *SWS2* mRNA with that of the wild-type (not *SWS2*-mutated) fish (*n* = 2) by stepwise RT–PCR. This experiment using eight adults was performed on two different genomic backgrounds, *ci* and Actb-SLα:GFP (i.e., *n* = 16 in total). No matter whether single or double, all the frameshift mutations commonly reduced the *sws2* transcripts on both backgrounds (Figures 2A,B), likely reflecting the nonsense-mediated mRNA decay (NMD) (Lykke-Andersen and Jensen, 2015). Thus, the *sws2* mutants should translate severely truncated SWS2 (Figure 1D) in small amounts (if any), which could further support the successful knockout of the *SWS2*.

Unexpectedly, *SWS2a* and *SWS2b* were expressed more strongly in Actb-SLα:GFP than they were in *ci* (Figure 2A). Until we noticed this, we repeated this assay without distinguishing the genomic backgrounds [as in our previous study for *LWS*s (Homma et al., 2017; Harada et al., 2019); note that *LWS* expressions in Figures 2A,B are similar between the backgrounds] being confused by apparently fluctuating or non-reproducible bands. Before we came to this conclusion, we had hypothesized the causes to include age (Valen et al., 2018), sex (Shao et al., 2014), and time of killing (Halstenberg et al., 2005). We might find a circadian fluctuation in *SWS2* expression (data not shown) but did not further assess genetic or environmental factors that could affect the *cone opsin* expression.

The *SWS2* expression in the standard wild-type strain that ordinarily expresses SLα (i.e., HNI) seemed to be more similar to that in *ci* than in Actb-SLα:GFP (Figure 2C). Thus, the *SWS2* expression seemed to be enhanced in Actb-SLα:GFP rather than suppressed in *ci* (discussed further below).

Expressional assays for the other *cone opsin* genes (Figure 2A) revealed that: (1) as *SWS2*s, *RH2c* is expressed more strongly in Actb-SLα:GFP than in *ci*; (2) the expression of *SWS1*, *RH2a*, *RH2b*, and *LWSa/b* (*LWSa* and *LWSb* are too similar to be analyzed separately) between *ci* and Actb-SLα:GFP is similar; and (3) the loss of blue opsin seems to not induce upregulation of violet, green, or red opsins on either genomic background.

### Mate Choice of the *sws2* Mutants

*ci* and Actb-SLα:GFP are body color variants of medaka (pale gray and dark orange, respectively, Fukamachi et al., 2004, 2009b) that strongly prefer to mate within the strain under white light when they were separately reared from hatching (Fukamachi et al., 2009a; Ikawa et al., 2017). These sexual preferences cannot be observed under monochromatic light (Utagawa et al., 2016) and are significantly weakened when the *LWS* genes are knocked out (Kamijo et al., 2018).

**FIGURE 3:**
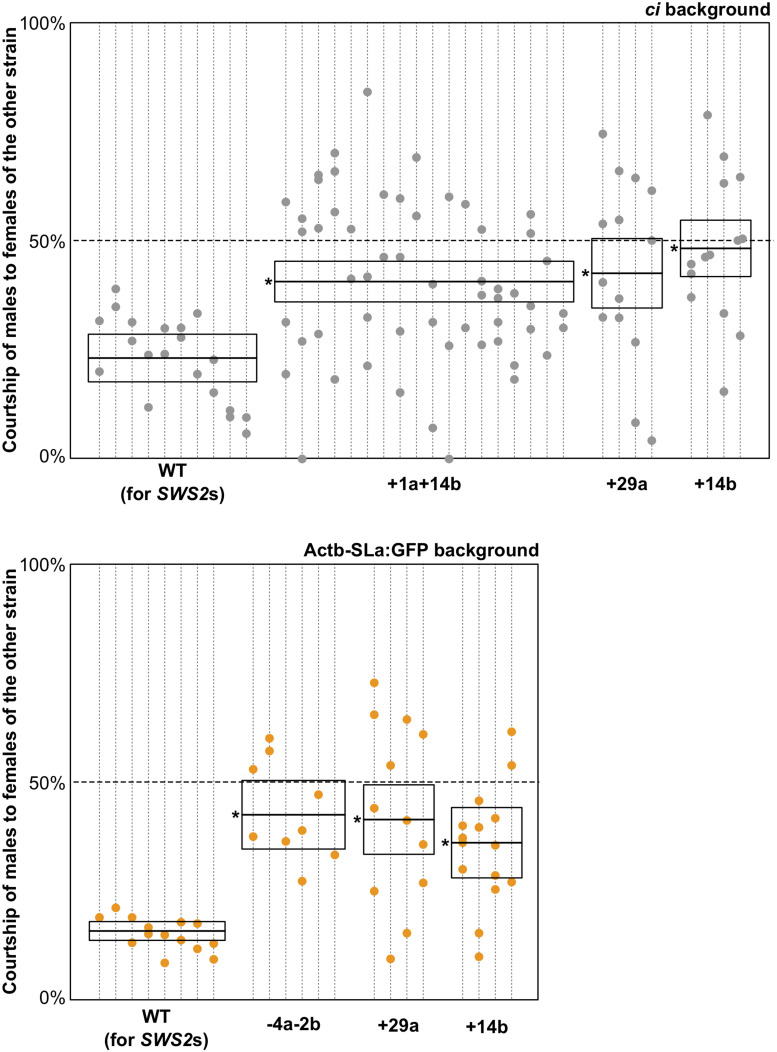
Mate choice of the *short-wavelength sensitive (sws)2* mutants. Male fish of wild type, *sws2*^+1a+14b^, *sws2^– 4a– 2b^*, *sws2*^+29a^, and *sws2*^+14b^ with the *color interfere* (*ci*) or actin beta (Actb)-somatolactin alpha (SLα):green fluorescent protein (GFP) background (shown by gray or orange, respectively) were given a choice between *ci* and Actb-SLα:GFP female fish. The *cone opsin* genotypes of the choice females were identical to those of the test males, except that we presented *ci* females with the *sws2*^+1a+14b^ mutation and Actb-SLα:GFP females with the *sws2*^+29a^ mutation to the wild-type males. Note that the wild-type males still exhibited strong preferences toward females of the same strain, indicating that body colors of the wild type and the *cone opsin* mutants are indistinguishable for not only humans but also medaka. Each dotted vertical line represents a male fish, and each circle on it is a result in a mate-choice trial (any trial with less than 10 courtships was ignored and is not shown). A box with a horizontal line shows the mean and 95% confidence interval of the ratio of courtship of male fish to female fish of the other strain (i.e., a relative sexual preference of the male fish). A one-way ANOVA followed by a Dunnett *post hoc* test revealed significant increases (shown by asterisks) in all the *sws2* mutants by comparison with the wild type (*P* < 0.05) on both genomic backgrounds.

Here, we similarly examined sexual preferences of the *SWS2*-mutated *ci* and Actb-SLα:GFP under white light (Figure 3). In terms of the wild-type (not *SWS2*-mutated) *ci* and Actb-SLα:GFP (*n* = 10 and 8, respectively), only 23.9% ± 6.5% and 15.7% ± 2.8% (mean ± 95% confidence interval) of male courtships were directed to female fish of the other strain, respectively. These proportions were significantly increased in the *sws2*-mutated *ci* and Actb-SLα:GFP (six strains in total). Namely, the proportions were 40.4% ± 5.1%, 42.3% ± 12.6%, and 48.0% ± 10.7% in the *sws2^+1a+14b^*, *sws2^+29a^*, and *sws2^+14b^* with the *ci* background (*n* = 18, 4, and 4, respectively) (*P* < 0.05, one-way ANOVA followed by a Dunnett *post hoc* test using the wild-type *ci* as a control) and 42.3% ± 10.5%, 41.2% ± 13.1%, and 35.9% ± 13.4% in the *sws2^–4a–2b^*, *sws2^+29a^*, and *sws2^+14b^* with the Actb-SLα:GFP background (*n* = 6, 4, and 4, respectively) (*P* < 0.05, one-way ANOVA followed by a Dunnett *post hoc* test using the wild-type Actb-SLα:GFP as a control). We note that all the mutant strains showed a common characteristic, i.e., whereas the wild-type male medaka never preferred female fish of the other strain, the mutant male fish did occasionally prefer the other strain. Thus, although the sample sizes per strain per background (particularly, those for the single mutants) were not very big, not only the *SWS2a/b*-double mutants (*n* = 24 in total) but also the single mutants (*n* = 16 in total) have weakened the body color preferences.

### Behavioral Photosensitivity of the *sws2* Mutants

As a lack of LWS clearly reduced sensitivity to red light (Homma et al., 2017; Matsuo et al., 2018), a lack of SWS2 may reduce sensitivity to blue light. Alternatively, the sensitivity may not be reduced because blue light could be absorbed by the neighboring violet and green opsins, whereas red (near infrared) light could not be absorbed by the neighboring green opsin. To address these hypotheses, we tested the blue light sensitivity of the *sws2* mutants. The OMR of medaka is so conspicuous that we could manually distinguish the wild-type and *lws*-mutant fish based on their behavior under monochromatic light at λ = 760 nm, i.e., all the 48 fish (of 77 with unknown genotype) we judged to be OMR-positive were indeed either *LWS*^+/+^ or *LWS*^+/–^ (Homma et al., 2017). That is, our manual assessments never included a false positive (but included false negatives because fish sometimes ignore/resist the rotating stripes).

Using the OLS (Watanabe et al., 1982), we performed the OMR test at wavelengths of every 10 nm between 380 and 450 nm (see Table 5 for photon flux density) using the wild type (*n* = 5) and the *sws2^–4a^*, *sws2^+8a^*, *sws2^+29a^*, *sws2^–8b^*, *sws2^+14b^*, *sws2^–4a–2b^*, and *sws2^–4a+2b^* mutants (*n* = 5, 5, 5, 5, 5, 3, and 1, respectively). For the wild type and the double mutants (*sws2^–4a–2b^* and *sws2^–4a+2b^*), we additionally tested at every 10 nm between 460 and 500 nm. In all the strains at all the wavelengths, we manually assessed the fish to be OMR-positive.

**TABLE 5:** Intensity of monochromatic light irradiated from the OLS.

Wavelength (nm)	Photon flux density (μmol/m^2^/s)
380	46
390	47
400	53
410	88
420	82
430	85
440	93
450	100
460	110
470	130
480	130
490	120
500	110

**OLS, Okazaki Large Spectrograph.**

**FIGURE 4:**
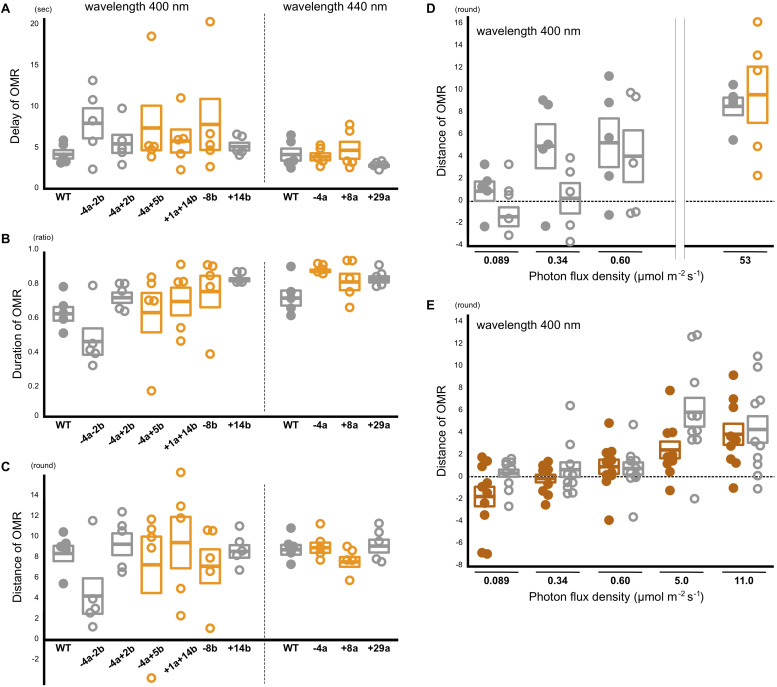
Behavioral photosensitivity of the *short-wavelength sensitive (sws)2* mutants. The optomotor response (OMR) of adult fish was tested individually under monochromatic light at λ = 400 nm for the wild type, *SWS2a/b*-double, and *SWS2b*-single mutants (left) and λ = 440 nm for the wild type and *SWS2a*-single mutants (right). Each circle represents the result for one fish (*n* = 5 for each strain). Closed circle, wild type; open circle, *sws2* mutants. Colors represent genomic backgrounds of the strains [gray, *color interfere* (*ci*); orange, actin beta (Actb)-somatolactin alpha (SLα):green fluorescent protein (GFP)]. The OMR was quantified by three parameters: **(A)** delay: the time elapsed until the fish started the OMR after switching the direction of stripe rotation, **(B)** duration: the proportion of time the fish was exhibiting the OMR, and **(C)** distance: the distance the fish swam in the direction of stripe rotation during the test (Matsuo et al., 2018). Mean and standard error of the mean is shown as a box with a horizontal line. No significant difference was detected between the wild type and the *sws2* mutants in any of the six comparisons (i.e., the delay, duration, or distance at λ = 400 or 440 nm) (*P* > 0.05; one-way ANOVA followed by a Dunnett *post hoc* test using the wild type as a control). **(D)** The OMR tests under mesopic conditions. The results at 53 μmol/m^2^/s (photopic condition) are those in **(C)**. Closed circle, wild type; open circle, *sws2*^+1a+14b^ (*n* = 5 each). See panel **(C)** for other symbols. The results at 0.089 μmol/m^2^/s (i.e., approximately –4 to + 4 rounds per individual and –2 to + 2 rounds in average) seem to indicate OMR-negative. From this standpoint, the OMR at 0.34 μmol/m^2^/s might be negative in the mutants, but positive in the wild type, although the difference is not significant (*P* > 0.05, Student’s *t*-test). **(E)** Further examination of the OMR under mesopic conditions. As the wild type (closed brown circles), we used the HNI strain (*n* = 9–10; one fish died during the tests). As the *sws2* mutants (open gray circles), we used the *sws2*^+1a+14b^ with the *ci* background (*n* = 10). The differences are not significant at any photon flux density (*P* > 0.05, Student *t* test). The results in panels **D,E** may be somewhat inconsistent (e.g., the response at 0.60 μmol/m^2^/s seems to be higher in **D** than it is in **E**), which would reflect, for example, experiments on different days using different fish, experiments at different times of the day (i.e., diurnal fluctuation of cone opsin expression), potential differences in light angles or rotation speeds, or deterioration of the xenon arc lamp [we adjusted the photon flux densities using neutral density filters and the slits of the Okazaki Large Spectrograph (OLS)].

The manual assessments might overlook quantitative reduction of the OMR in the *sws2* mutants. Thus, we additionally performed quantitative OMR tests (Matsuo et al., 2018) focusing on λ = 440 nm for the *SWS2a*-single mutants (*sws2^–4a^*, *sws2^+8a^*, and *sws2^+29a^*; *n* = 5 each) and λ = 400 nm for the *SWS2b*-single mutants (*sws2^–8b^* and *sws2^+14b^*; *n* = 5 each) and the *SWS2a/b*-double mutants (*sws2^+1a+14b^*, *sws2^–4a–2b^*, and *sws2^–4a+2b^*; *n* = 5 each). We chose these wavelengths because λ_max_ of SWS2a and SWS2b are 439 and 405 nm, respectively, and those of the neighboring SWS1 and RH2a are 356 and 452 nm, respectively (Matsumoto et al., 2006). Their genomic backgrounds were *ci* for *sws2^+29a^*, *sws2^+14b^*, *sws2^–4a–2b^*, and *sws2^–4a+2b^* and Actb-SLα:GFP for *sws2^–4a^*, *sws2^+8a^*, *sws2^–8b^*, *sws2^–4a+5b^*, and *sws2^+1a+14b^*. We used *ci* as the wild-type control (*n* = 5). As shown in Figures 4A–C, all the *sws2* mutants responded to the rotating stripes as quickly (Figure 4A) and for as long (Figure 4B) as the wild type, which resulted in the similar overall swimming distances per OMR test (Figure 4C; *P* > 0.05, one-way ANOVA followed by a Dunnett *post hoc* test).

We further hypothesized that a difference in the OMR (i.e., blue light sensitivity) could not be detected in the experiments described above because the monochromatic light from the OLS was too strong (i.e., bright enough even for the *sws2* mutants to fully recognize the rotating stripes). Thus, we performed the quantitative OMR tests at decreased photon flux densities (i.e., at mesopic conditions) using the wild type and the *sws2^+1a+14b^* mutants with the *ci* background (*n* = 5 each). Whereas both the strains swam for about nine rounds per OMR test at 53 μmol/m^2^/s (Figures 4C,D), the distance dropped to four to five rounds at 0.60 μmol/m^2^/s in both strains. At 0.089 μmol/m^2^/s, the OMR seemed to be substantially negative in both strains (i.e., 1.1 ± 0.9 and –1.2 ± 0.9 rounds, respectively; it should be noted that these are distances for which fish followed the rotating stripes in 2 min in a tank of 19 cm diameter; see section “Materials and Methods”). At 0.34 μmol/m^2^/s, the wild type might better respond than the *sws2^+1a+14b^* mutant, although the difference was not significant (*P* = 0.095, Student’s *t*-test).

To assess further the potential difference in behavioral photosensitivity under the mesopic condition, we repeated the OMR tests using different individuals. We used the standard HNI strain (*n* = 10) and the *sws2^+1a+14b^* mutant with the *ci* background (*n* = 10; Figure 4E). The OMR seems to be positive at 5.0 and 11.0 μmol/m^2^/s and negative at 0.089 μmol/m^2^/s in both strains. At neither 0.34 nor 0.60 μmol/m^2^/s an apparent or statistically significant difference in the OMR could be detected (*P* > 0.05, Student’s *t-*test). A difference might be detected at different photon flux densities or wavelengths or by methods other than the OMR test. However, effects of the SWS2 loss on behavioral blue light sensitivity would in any case be subtle, or might even be absent, while the defects in color-dependent mate choice were relatively apparent (Figure 3).

## Discussion

### Apparent Effects of the *sws2* Mutations on Mate Choice but Not the Optomotor Response

Although we did not confirm it at the protein level (antibodies specific for the medaka SWS2a or SWS2b are not available), the frameshift mutations (Tables 1–3 and Figure 1D) and the suppressed mRNA expressions (Figures 2A,B) strongly suggest that the *SWS2a/b*-double mutants lack functional blue opsins. On losing one of the four types of cone opsins in the retina, the mutants had been expected to have a defect in color vision, and reduced body color preferences were actually detected (Figure 3). The similar reduction in body color preferences had also been observed in the *LWSa/b*-double mutants (Kamijo et al., 2018). Therefore, we suspect that these cone opsin knockouts could not distinguish the body colors of *ci* and Actb-SLα:GFP as clearly as the wild type, and this visual limitation caused the weakly biased color preferences, as reported in other fish using turbid water (Engström-Öst and Candolin, 2007; Sundin et al., 2010; Ehlman et al., 2018). To know to what extent the abilities for color discrimination are actually reduced in the *sws2* and *lws* mutants, further experiments using different colors, possibly based on learning (e.g., Siebeck et al., 2014; Escobar-Camacho et al., 2017), are necessary.

Four types of cone cells are regularly arranged in the fish retina forming the retinal mosaic (Nishiwaki et al., 1997; Allison et al., 2010), but expressions of cone opsins are rather complex; for example, different types of opsins could be coexpressed in a single cone and the expression can change relying on growth stages, ambient light, or visual angles (Dalton et al., 2014; Sakai et al., 2018; Zimmermann et al., 2018). In our studies, proximate consequences of the SWS2 or LWS loss (e.g., effects on the retinal mosaic or electrophysiological responses in the retina or downstream cascades) remain unknown, which are inevitable (and complex) subjects for unveiling the mechanism for color perception (Thoreson and Dacey, 2019). However, one solid conclusion at present is that our reverse-genetic studies demonstrated causal relationships (not simply associations) between a cone opsin repertoire and a color-dependent social behavior (Figure 3; Kamijo et al., 2018).

Despite the apparent effects on mate choice, blue light sensitivity of the *sws2* mutants was equivalent to that of the wild type (Figure 4). By contrast, in our previous study, an apparent reduction in photopic red light sensitivity could be detected in the *lws* mutants (Homma et al., 2017; Matsuo et al., 2018). These potentially controversial results are not at all surprising because whereas red light (λ > 740 nm) could only be absorbed by LWSs (λ_max_ = 561–562 nm), blue light tested in this study (λ = 380–500 nm) could likely be absorbed by not only SWS2s (λ_max_ = 405–439 nm) but also the neighboring SWS1 (λ_max_ = 356 nm) and RH2s (λ_max_ = 452–516 nm) (Matsumoto et al., 2006). Indeed, we previously found that the wild-type and *LWSa/b*-knockout medaka could exhibit the OMR at λ ≤ 830 and ≤ 740 nm, respectively (Homma et al., 2017; Matsuo et al., 2018). That is, LWSs and RH2s can absorb and make medaka respond behaviorally to light at wavelengths > 200 nm longer than the λ_max_. The absorption of a wide range of light might be the same for SWS2s and SWS1, which enabled compensation of the SWS2 loss by SWS1 (and maybe also RH2 and LWS, although we still do not know how widely the cone opsins cover wavelengths shorter than the λ_max_). The present results also indicate that the compensation does not require upregulation of *SWS1*, *RH2*, or *LWS* (Figure 2A). Compensation by RH1 is unlikely because the rod vision has been shown to be dysfunctional in light-adapted medaka, i.e., only the dark-adapted, but not light-adapted, *lws* mutants could show the OMR at λ ≥ 750 nm (Homma et al., 2017).

We note that SWS2 also plays only a dispensable role for blue light sensitivity in zebrafish larvae, i.e., when the *SWS2*-expressing cones are chemically ablated, the ability to detect contrasts between dark red and blue on an RGB monitor is temporarily reduced, but quickly recovered within 24 h without regenerating the *SWS2* cones (Hagerman et al., 2016). Thus, the absence of SWS2 seems to have little effect on photopic blue light sensitivity in both medaka adults and zebrafish larvae.

### Adaptive Evolution of Cone Opsin Repertoire

Divergent repertoires and λ_max_ of cone opsins in fish have often been discussed in relation to adaptation to various underwater light conditions and body colors of mating partners; for example, in blue light-dominating clear water, blue light sensitivity is increased by increased *SWS2* expression or blue-shift of λ_max_, which promotes sexual selection of a blue nuptial coloration. Although this evolutionary scenario (i.e., sensory drive) sounds attractive and many researchers are reporting results that support it (see Cummings and Endler, 2018 and references therein), the reverse-genetic evidence in medaka and zebrafish demonstrating little difference in behavioral blue light sensitivity regardless of the presence or absence of SWS2 even at a wavelength close to its λ_max_ (Figure 4; Hagerman et al., 2016) seems to suggest that the “dominant-light” hypothesis needs to be reconsidered carefully.

Increased photosensitivity to a dominating wavelength should increase not only a signal from an object of interest but also noise from the background. A study reporting a depth-dependent shift in λ_max_ of SWS2, but away from a dominating wavelength in cottoid fishes (whereas RH1 for scotopic vision did shift toward the dominating wavelength) (Cowing et al., 2002), is implicative in that the authors suggested that the shift is to increase the signal-to-noise ratio (the “noise reduction” hypothesis). In Catarrhini, the functional importance of the duplicated and diversified λ_max_ in LWSs is theoretically (Lewis and Li, 2006) and ecologically (Melin et al., 2017) interpreted for discrimination of red objects (e.g., fruits) against green background (e.g., leaves). Animals other than Catarrhini should also have a particular color (or luminance) that needs to be discriminated against background for efficient survival and reproduction in nature, which could drive the diversifications of repertoire, λ_max_, or expression of cone opsins (the “color discrimination” hypothesis).

### Plastic Expression of the *Cone Opsin* Genes

Another interesting finding in this study is the apparent increase in the expression of *SWS2a*, *SWS2b*, and *RH2c* in Actb-SLα:GFP in comparison with that in *ci* (Figure 2). The expression of cone opsins is plastic depending on, for example, light conditions (Fuller et al., 2010; Hofmann et al., 2010; Sakai et al., 2016) and circadian rhythms (Halstenberg et al., 2005; Li, 2005; Johnson et al., 2013), but all the fish used in Figure 2 were reared under identical light conditions and killed for RNA extraction at the same time. The Actb-SLα:GFP strain is established by introducing a transgene that expresses SLα and *Renilla* GFP (hrGFP II; Agilent Technologies) into *ci* (Fukamachi et al., 2009b). Therefore, nothing else but SLα and/or *Renilla* GFP must be the cause for the differences in expression of *SWS2a*, *SWS2b*, and *RH2c* between *ci* and Actb-SLα:GFP.

**FIGURE 5:**
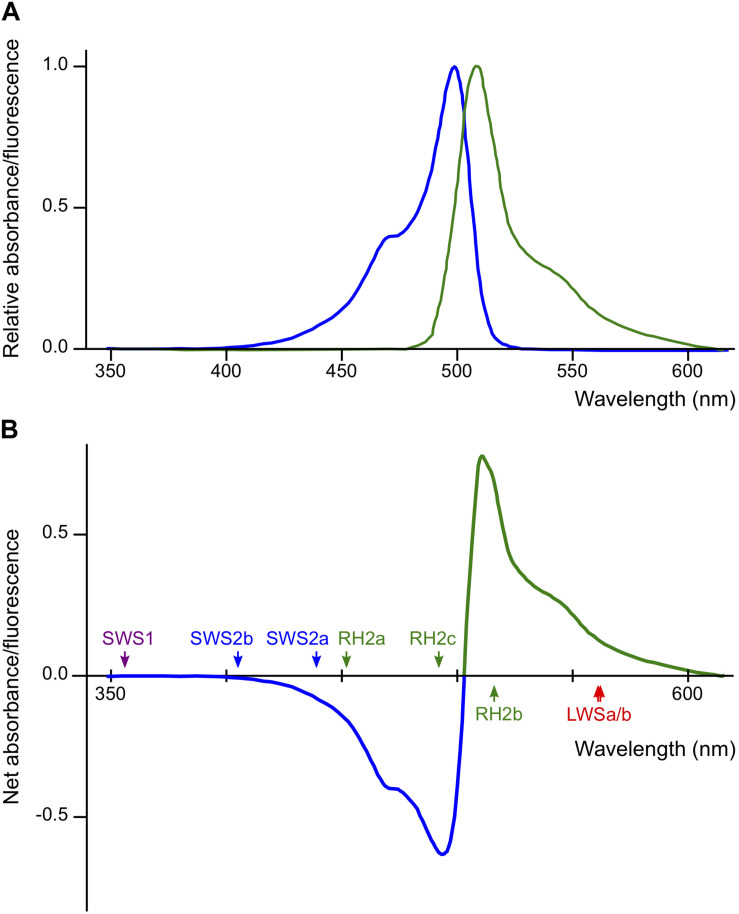
Potential effects of *Renilla* green fluorescent protein (GFP) on the vision of actin beta (Actb)-somatolactin alpha (SLα):GFP. **(A)** Excitation (blue) and emission (green) spectra of *Renilla* GFP (hrGFPII; Agilent Technologies) (Ward and Cormier, 1979). Excitation and emission peaks are 500 and 506 nm, respectively. The spectra are largely symmetrical using 503 nm as a border. **(B)** Net spectral absorbance (minus values) and fluorescence (plus values) by *Renilla* GFP based on the data in **(A)**. This graph indicates that a part of the blue light (λ < 503 nm) impinging on the eyes of Actb-SLα:GFP (through the cornea and lens that weakly express *Renilla* GFP; Fukamachi et al., 2009b) would be absorbed, converted to green light (λ > 503 nm), and scattered into all directions before reaching the retina. The λ_max_ of the medaka cone opsins are shown by colored arrows.

The *Renilla* GFP in Actb-SLα:GFP is not visible under white light. However, it does exist in all cells that express the gene for Actb, including in the lenses of the eyes (Fukamachi et al., 2009b). According to the datasheet provided by the manufacturer, absorption/emission spectra of *Renilla* GFP are nearly symmetrical with absorption/emission peaks at 500/506 nm, respectively (Figure 5A). This indicates that, using 503 nm as a border, a part of light at shorter wavelengths (blue light) is absorbed and converted to light at longer wavelengths (green light) before reaching the retina of Actb-SLα:GFP (Figure 5B). Some of the green light would directly go out of the eyes because the direction of emission by *Renilla* GFP molecules must be random. Thus, the retina of Actb-SLα:GFP would mostly receive less blue light (and more green light) in comparison with that of *ci*, i.e., *ci* and Actb-SLα:GFP should effectively live in different light conditions.

However, again, this interpretation becomes inconsistent with the “dominant-light” hypothesis because λ_max_ of the increased *SWS2a*, *SWS2b*, and *RH2c* (439, 405, and 492 nm, respectively) are within the range of decreased ambient light (λ = 400–500 nm). We interpreted this result that the decreased blue light impinging on the retina (decreased signals from blue light-absorbing cone cells) was compensated for by the increased expression of *SWS2a*, *SWS2b*, and *RH2c*, i.e., the biased light spectrum was corrected by counter-biased expression of the cone opsins (the “white-balance” hypothesis). However, this interpretation does not explain the lack of increase in *RH2a* with λ_max_ at 452 nm in Actb-SLα:GFP (Figure 2A).

An alternative interpretation is that SLα directly (or indirectly *via* body color recognition; Ikawa et al., 2017) enhanced the *SWS2a*/*SWS2b*/*RH2c* expression. Considering that the expression of *SWS2*s between SLα-expressing HNI and SLα-deficient *ci* (Figure 2C) is similar, *Renilla* GFP would more likely be the cause. Reverse genetics to remove the *Renilla GFP* from Actb-SLα:GFP or rearing *ci* in *Renilla* GFP solution, for example, may address these possibilities.

## Conclusion

The present study established *SWS2*-knockout medaka and demonstrated a defect in color-dependent mating behaviors (Figure 3). However, its defect in behavioral blue light sensitivity is subtle, if not absent (Figure 4). These results could possibly be interpreted that, whereas a signal from the SWS2-expressing cones (together with those from the SWS1/RH2/LWS-expressing cones, i.e., a signal of four channels) is essential for the normal (tetrachromatic) color vision, the blue channel is dispensable and the remaining violet/green/red channels are sufficient for detecting blue light (although the three-channel signal may not be sensed as blue). This kind of a clear-cut evidence demonstrating a causal relationship (not an association) between genotype and phenotype can only be obtained by reverse (or forward) genetics. The recent genome-editing technologies have brought animal-vision studies to a new era. Although the diversification in cone opsins has often exclusively been discussed in relation to the sensory-drive hypothesis, the present results strongly suggest that increased transcription or even acquiring cone opsin types/subtypes does not necessarily increase (i.e., adapt) behavioral photosensitivity to a certain wavelength in photopic or mesopic conditions. Other viewpoints are necessary to understand the evolution of color vision in animals.

## Materials and Methods

We essentially adopted the same procedures which were used to establish and characterize the *lws* mutants (Homma et al., 2017; Kamijo et al., 2018; Matsuo et al., 2018; Harada et al., 2019). Here, we outline the strategies in brief.

### Targeted Mutagenesis of the *SWS2* Genes by the CRISPR/Cas9 System

The *ci* and Actb-SLα:GFP strains were used as hosts for genome editing. *ci* has a mutation on the gene for SLα (Fukamachi et al., 2004), and Actb-SLα:GFP is a transgenic *ci* strain that ectopically overexpresses SLα driven by the promotor for *actin beta* (*Actb*) (Fukamachi et al., 2009b). The transgene also expresses *Renilla* GFP under the control of the internal ribosome entry site (IRES). We have been using these strains to establish color-blind medaka because of their unique color-dependent mate-choice behaviors (Fukamachi et al., 2009a; Utagawa et al., 2016; Ikawa et al., 2017).

Double-strand oligonucleotides complementary to target sequences (5′-CCTGTCTGTGGCTAATCTTCT-3′ for *SWS2a* and 5′-ACATGGTTCTGGGTCCACTGG-3′ for *SWS2b*) were inserted into a DR274 vector (Addgene) from which guide RNA (gRNA) were transcribed using an AmpliScribe T7-flash Transcription Kit (Epicentre). The *Cas9* mRNA was synthesized from an hCas9 vector (Addgene) using an mMessage mMachine SP6 kit (Life Technology). The RNAs were purified using an RNeasy mini Kit (Qiagen) and microinjected into one-cell-stage embryos using GD-1 glass capillaries (Narishige) and an IM-9b microinjector (Narishige) under an SZX16 stereomicroscope (Olympus).

Ins/del mutations induced on the target sequences were detected using a heteroduplex mobility assay (HMA) on 12% polyacrylamide gels using the primers shown in Table 6. The microinjected adults that possess an ins/del mutation (G_0_) were crossed with the wild type to obtain heterozygotes of the ins/del mutation (F_1_). Ins/del mutations causing a frameshift were screened by genomic PCR and direct sequencing using the primers shown in Table 6. Heterozygous fish with an identical frameshift mutation on *SWS2a* and/or *SWS2b* were intercrossed to obtain homozygotes for the mutation/s.

**TABLE 6:** A list of primers used in this study.

Gene	Purpose	Sequence (5′–3′)
*SWS1*	RT-PCR	f: ATGGGAAAATACTTCTACCTGTATGAGAACATC
		r: TTAAGAGGCCGTGGACACCTCCG
*SWS2a*	HMA	f: AACAAGAAGCTTCGATCCCA
		r: ATATCTGCAAGCGAAGGAGC
	Direct sequencing	f: TCATCAGTGGTGGGGAGCTG
		r: AAAGTTWCCAAGYGGCTTGCAGA*
	RT-PCR	f: TCATCAGTGGTGGGGAGCTG
		r: CTAAGCTGGTCCGACTTTAGAGACTTC
*SWS2b*	HMA	f: TTGTTGCTTCTACGGGTTCC
		r: TTTGGCTCTAGAGAGGTACAGTCA
	Direct sequencing	f: GGGGAAATCGTGTTGTGGAGTTT
		r: AAAGTTWCCAAGYGGCTTGCAGA*
	RT-PCR	f: GGGGAAATCGTGTTGTGGAGTTT
		r: TTAGGAAGGGCCGACTTTTGAGACTTC
*RH2a*	RT-PCR	f: ATGGAGAACGGCACAGAGGGCAAG
		r: CAAGCAGCAGTAGAGACTTCTGTCTTGC
*RH2b*	RT-PCR	f: GGGTTGGGAGCCTAATGGCACTG
		r: GAGGTTGTTGTAATTAAGACATATGGTCCT**
*RH2c*	RT-PCR	f: ATGGGCTGGGATGGAGGAGAGC
		r: GAGGTTGTTGTAATTAAGACATATGGTCCT**
LWSa/b***	RT-PCR	f: GGCAGAGSAGTGGGGAAAACAGG
		r: TATGCAGGAGCCACAGAGGAGACC

*These primer sequences are identical. **These primer sequences are identical. ***Sequences of LWSa and LWSb are too similar to be amplified separately. HMA, heteroduplex mobility assay; LWS, long-wavelength sensitive; RH, rhodopsin; SWS, short-wavelength sensitive.

### Reverse Transcription Polymerase Chain Reaction

Total RNA was extracted from the eyes of fully matured adults using Isogen II reagent (Nippon Gene), incubated with deoxyribonuclease (RT Grade) for Heat Stop (Nippon Gene), and used as templates for reverse transcription by ReverTra Ace (Toyobo) and polyT primers. PCR primers for each *cone opsin* gene are listed in Table 6. Temperature conditions were: 96°C for 1 min; 20–30 cycles (we performed stepwise PCR for each gene and stopped the reaction before the amplification plateaued) of 98°C for 20 s, 60°C for 1 min, 72°C for 1 min; and 72°C for 10 min. The products were electrophoresed on a 1% agarose gel and detected by ethidium bromide staining and UV transillumination (BioDoc-It Imaging System, UVP).

### Mate-Choice Experiments

The medaka spawn every morning. We put one male and two female fish in a tank (20 cm × 13 cm with a water level of about 5 cm), let them mate freely for 30 min, and manually counted courtships (approaching behaviors) to each female fish. The body length was strictly equalized between choice females; i.e., the difference was less than 1 mm. A sexual preference of the male fish in the trial was calculated as a ratio of the courtships. If a male fish courted less than 10 times in a trial, we abolished the datum. This trial was repeated for two or four times in consecutive 2 or 4 days presenting different female individuals, and an overall preference of the male was calculated as an average in the trials. A preference of a strain was calculated as an average of all the male fish in the strain, and the values were compared between strains by a one-way analysis of variance (ANOVA) followed by a Dunnett *post hoc* test using IBM SPSS Statistics (ver. 25) for Mac (IBM Corp.).

### The Optomotor Response Test

Bright-adapted adult medaka were put into a cylindrical glass tank (19 cm in diameter). The tank was placed in a drum (24 cm in diameter), inside of which vertical stripes were made using Indian ink-painted plastic paper and aluminum foil (because ordinary white paper and black ink fluoresce under blue light). The entire drum was irradiated from the top using monochromatic light from an OLS (Watanabe et al., 1982). In the experiments in Figures 4D,E, we simultaneously irradiated infrared light (λ = 940 nm) using Hololight (PiPhotonics) because the blue light was not sufficiently strong enough for video recording. This infrared light should not affect the OMR (i.e., should be invisible for medaka) because the OMR was negative at λ > 840 nm in our previous experiments (Homma et al., 2017; Matsuo et al., 2018).

After 30 s of acclimation, the stripes were rotated in the clockwise, counterclockwise, clockwise, and counterclockwise directions in 10 rpm for 30 s each. Behaviors were video-recorded using an A10FHDIR (Kenko) or an ORCA-R2 digital CCD camera (Hamamatsu Photonics). Because the ORCA-R2 camera was not very sensitive to light at λ < 400 nm, some image processing was needed to analyze the movies recorded at λ = 400 nm, which are summarized in Supplementary Movie S1.

The position (x–y coordinates) of fish in each video frame was extracted using UMATracker software (Yamanaka and Takeuchi, 2018), from which we calculated three parameters to quantify the OMR: delay, duration, and distance (Matsuo et al., 2018). The delay and duration were calculated as averages, whereas the distance was a sum, in four rotations. The values in each parameter were compared at each wavelength using a one-way ANOVA followed by a Dunnett *post hoc* test using the wild type as a control (Figures 4A–C). For Figures 4D,E, we applied a Student’s *t*-test at each photon flux density.

## Data Availability Statement

Strains and plasmids are available upon request. The authors affirm that all data necessary for confirming the conclusions of the article are present within the article, figures, and tables.

## Ethics Statement

The animal study was reviewed and approved by the Animal Experiment Committee of Japan Women’s University.

## Author Contributions

YH introduced the *sws2* mutations. NK and MG established and characterized the *sws2* mutants (the OMR and RT-PCR). CT and YS performed the mate choice. MM quantitatively analyzed the OMR. TU and YK provided technical advice on the OLS. SF examined the *SWS2* loci *in silico*, designed and supervised the wet experiments, and wrote the manuscript. All authors contributed to the article and approved the submitted version.

## Conflict of Interest

The authors declare that the research was conducted in the absence of any commercial or financial relationships that could be construed as a potential conflict of interest.

## Footnotes

1 http://utgenome.org/medaka_v2/#!Top.md

2 https://shigen.nig.ac.jp/medaka/

## Supplementary Material

MOVIE S1 Summary of retouch processes for tracking movies recorded at λ = 400 nm.

## References

[B1] Allison T. W. Barthel L. K. Skebo K. M. Takechi M. Kawamura S. Raymond P. A. (2010). Ontogeny of cone photoreceptor mosaics in zebrafish. *J. Comp. Neurol.* 518 4182–4195. 10.1002/cne.22447 20878782 PMC3376642

[B2] Bowmaker J. K. (1998). Evolution of colour vision in vertebrates. *Eye* 12 541–547. 10.1038/eye.1998.143 9775215

[B3] Carleton K. L. Escobar-Camacho D. Stieb S. M. Cortesi F. Marshall N. J. (2020). Seeing the rainbow: mechanisms underlying spectral sensitivity in teleost fishes. *J. Exp. Biol.* 223:jeb193334. 10.1242/jeb.193334 32327561 PMC7188444

[B4] Conway B. R. (2009). Color vision, cones, and color-coding in the cortex. *Neuroscientist* 15 274–290. 10.1177/1073858408331369 19436076

[B5] Cortesi F. Musilová Z. Stieb S. M. Hart N. S. Siebeck U. E. Malmstrøm M. (2015). Ancestral duplications and highly dynamic opsin gene evolution in percomorph fishes. *Proc. Natl. Acad. Sci. U.S.A.* 112 1493–1498. 10.1073/pnas.1417803112 25548152 PMC4321309

[B6] Cowing J. A. Poopalasundaram S. Wilkie S. E. Bowmaker J. K. Hunt D. M. (2002). Spectral tuning and evolution of short wave-sensitive cone pigments in cottoid fish from Lake Baikal. *Biochemistry* 41 6019–6025. 10.1021/bi025656e 11993996

[B7] Cummings M. E. Endler J. A. (2018). 25 Years of sensory drive: the evidence and its watery bias. *Curr. Zool.* 64 471–484. 10.1093/cz/zoy043 30108628 PMC6084598

[B8] Dalton B. E. Loew E. R. Cronin T. W. Carleton K. L. (2014). Spectral tuning by opsin coexpression in retinal regions that view different parts of the visual field. *Proc. R. Soc. B Biol. Sci.* 281 1–9. 10.1098/rspb.2014.1980 25377457 PMC4240992

[B9] Ehlman S. M. Martinez D. Sih A. (2018). Male guppies compensate for lost time when mating in turbid water. *Behav. Ecol. Sociobiol.* 72:46 10.1007/s00265-018-2468-8

[B10] Engström-Öst J. Candolin U. (2007). Human-induced water turbidity alters selection on sexual displays in sticklebacks. *Behav. Ecol.* 18 393–398. 10.1093/beheco/arl097 27193460

[B11] Escobar-Camacho D. Marshall J. Carleton K. L. (2017). Behavioral color vision in a cichlid fish: metriaclima benetos. *J. Exp. Biol.* 220 2887–2899. 10.1242/jeb.160473 28546509 PMC5576065

[B12] Fukamachi S. Kinoshita M. Aizawa K. Oda S. Meyer A. Mitani H. (2009a). Dual control by a single gene of secondary sexual characters and mating preferences in medaka. *BMC Biol.* 7:64. 10.1186/1741-7007-7-64 19788724 PMC2761876

[B13] Fukamachi S. Yada T. Meyer A. Kinoshita M. (2009b). Effects of constitutive expression of somatolactin alpha on skin pigmentation in medaka. *Gene* 442 81–87. 10.1016/j.gene.2009.04.010 19393303

[B14] Fukamachi S. Sugimoto M. Mitani H. Shima A. (2004). Somatolactin selectively regulates proliferation and morphogenesis of neural-crest derived pigment cells in medaka. *Proc. Natl. Acad. Sci. U.S.A.* 101 10661–10666. 10.1073/pnas.0401278101 15249680 PMC489991

[B15] Fuller R. C. Noa L. A. Strellner R. S. (2010). Teasing apart the many effects of lighting environment on opsin expression and foraging preference in bluefin killifish. *Am. Nat.* 176 1–13. 10.1086/652994 20497054

[B16] Greenwald S. H. Kuchenbecker J. A. Roberson D. K. Neitz M. Neitz J. (2014). S-opsin knockout mice with the endogenous M-opsin gene replaced by an L-opsin variant. *Vis. Neurosci.* 31 25–37. 10.1017/S0952523813000515 24801621 PMC4167788

[B17] Hagerman G. F. Noel N. C. L. Cao S. Y. DuVal M. G. Oel A. P. Allison W. T. (2016). Rapid recovery of visual function associated with blue cone ablation in Zebrafish. *PLoS One* 11:e0166932. 10.1371/journal.pone.0166932 27893779 PMC5125653

[B18] Halstenberg S. Lindgren K. M. Samagh S. P. S. Nadal-Vicens M. Balt S. Fernald R. D. (2005). Diurnal rhythm of cone opsin expression in the teleost fish Haplochromis burtoni. *Vis. Neurosci.* 22 135–141. 10.1017/S0952523805222022 15935106

[B19] Harada Y. Matsuo M. Kamei Y. Goto M. Fukamachi S. (2019). Evolutionary history of the medaka long-wavelength sensitive genes and effects of artificial regression by gene loss on behavioural photosensitivity. *Sci. Rep.* 9 1–11. 10.1038/s41598-019-39978-6 30804415 PMC6389941

[B20] Hering E. (1920). *Grundzüge der Lehre Vom Lichtsinn.* Berlin: Springer.

[B21] Hofmann C. M. O’Quin K. E. Smith A. R. Carleton K. L. (2010). Plasticity of opsin gene expression in cichlids from Lake Malawi. *Mol. Ecol.* 19 2064–2074. 10.1111/j.1365-294X.2010.04621.x 20374487

[B22] Homma N. Harada Y. Uchikawa T. Kamei Y. Fukamachi S. (2017). Protanopia (red color-blindness) in medaka: a simple system for producing color-blind fish and testing their spectral sensitivity. *BMC Genet.* 18:10. 10.1186/s12863-017-0477-7 28166717 PMC5294709

[B23] Ikawa M. Ohya E. Shimada H. Kamijo M. Fukamachi S. (2017). Establishment and maintenance of sexual preferences that cause a reproductive isolation between medaka strains in close association. *Biol. Open* 6 244–251. 10.1242/bio.022285 28202469 PMC5312102

[B24] Johnson A. M. Stanis S. Fuller R. C. (2013). Diurnal lighting patterns and habitat alter opsin expression and colour preferences in a killifish. *Proc. R. Soc. B Biol. Sci.* 280:20130796. 10.1098/rspb.2013.0796 23698009 PMC3774230

[B25] Kamijo M. Kawamura M. Fukamachi S. (2018). Loss of red opsin genes relaxes sexual isolation between skin-colour variants of medaka. *Behav. Processes* 150 25–28. 10.1016/j.beproc.2018.02.006 29447852

[B26] Lewis A. Li Z. (2006). Are cone sensitivities determined by natural color statistics? *J. Vis.* 6 285–302. 10.1167/6.3.8 16643096

[B27] Li P. (2005). Circadian rhythms of behavioral cone sensitivity and long wavelength opsin mRNA expression: a correlation study in zebrafish. *J. Exp. Biol.* 208 497–504. 10.1242/jeb.01424 15671338

[B28] Lykke-Andersen S. Jensen T. H. (2015). Nonsense-mediated mRNA decay: an intricate machinery that shapes transcriptomes. *Nat. Rev. Mol. Cell Biol.* 16 665–677. 10.1038/nrm4063 26397022

[B29] Marques D. A. Taylor J. S. Jones F. C. Di Palma F. Kingsley D. M. Reimchen T. E. (2017). Convergent evolution of SWS2 opsin facilitates adaptive radiation of threespine stickleback into different light environments. *PLoS Biol.* 15:e2001627. 10.1371/journal.pbio.2001627 28399148 PMC5388470

[B30] Matsumoto Y. Fukamachi S. Mitani H. Kawamura S. (2006). Functional characterization of visual opsin repertoire in Medaka (*Oryzias latipes*). *Gene* 371 268–278. 10.1016/j.gene.2005.12.005 16460888

[B31] Matsuo M. Ando Y. Kamei Y. Fukamachi S. (2018). A semi-automatic and quantitative method to evaluate behavioral photosensitivity in animals based on the optomotor response (OMR). *Biol. Open* 7:bio033175. 10.1242/bio.033175 29921705 PMC6031347

[B32] Melin A. D. Chiou K. L. Walco E. R. Bergstrom M. L. Kawamura S. Fedigan L. M. (2017). Trichromacy increases fruit intake rates of wild capuchins (*Cebus capucinus* imitator). *Proc. Natl. Acad. Sci. U.S.A.* 114 10402–10407. 10.1073/pnas.1705957114 28894009 PMC5625910

[B33] Neitz J. Neitz M. (2011). The genetics of normal and defective color vision. *Vision Res.* 51 633–651. 10.1016/j.visres.2010.12.002 21167193 PMC3075382

[B34] Neitz J. Neitz M. He J. C. Shevell S. K. (1999). Trichromatic color vision with only two spectrally distinct photopigments. *Nat. Neurosci.* 2 884–888. 10.1038/13185 10491608

[B35] Neitz M. Neitz J. (2014). Curing color blindness—mice and nonhuman primates. *Cold Spring Harb. Perspect. Med.* 4:a017418. 10.1101/cshperspect.a017418 25147187 PMC4208712

[B36] Nishiwaki Y. Oishi T. Tokunaga F. Morita T. (1997). Three-dimensional reconstitution of cone arrangement on the spherical surface of the retina in the medaka eyes. *Zoolog. Sci.* 14 795–801. 10.2108/zsj.14.79

[B37] Sakai Y. Kawamura S. Kawata M. (2018). Genetic and plastic variation in opsin gene expression, light sensitivity, and female response to visual signals in the guppy. *Proc. Natl. Acad. Sci. U.S.A.* 115 12247–12252. 10.1073/pnas.1706730115 30420507 PMC6275514

[B38] Sakai Y. Ohtsuki H. Kasagi S. Kawamura S. Kawata M. (2016). Effects of light environment during growth on the expression of cone opsin genes and behavioral spectral sensitivities in guppies (*Poecilia reticulata*). *BMC Evol. Biol.* 16:106. 10.1186/s12862-016-0679-z 27193604 PMC4870739

[B39] Shao Y. T. Wang F. Y. Fu W. C. Yan H. Y. Anraku K. Chen I. S. (2014). Androgens increase lws opsin expression and red sensitivity in male three-spined sticklebacks. *PLoS One* 9:e100330. 10.1371/journal.pone.0100330 24963891 PMC4070989

[B40] Siebeck U. E. Wallis G. M. Litherland L. Ganeshina O. Vorobyev M. (2014). Spectral and spatial selectivity of luminance vision in reef fish. *Front. Neural Circuits* 8:118. 10.3389/fncir.2014.00118 25324727 PMC4179750

[B41] Solomon S. G. Lennie P. (2007). The machinery of colour vision. *Nat. Rev. Neurosci.* 8 276–286. 10.1038/nrn2094 17375040

[B42] Sundin J. Berglund A. Rosenqvist G. (2010). Turbidity hampers mate choice in a pipefish. *Ethology* 116 713–721. 10.1111/j.1439-0310.2010.01787.x

[B43] Taylor M. R. Kikkawa S. Diez-Juan A. Ramamurthy V. Kawakami K. Carmeliet P. (2005). The zebrafish pob gene encodes a novel protein required for survival of red cone photoreceptor cells. *Genetics* 170 263–273. 10.1534/genetics.104.036434 15716502 PMC1449739

[B44] Thanaraj T. A. (2001). Human GC-AG alternative intron isoforms with weak donor sites show enhanced consensus at acceptor exon positions. *Nucleic Acids Res.* 29 2581–2593. 10.1093/nar/29.12.2581 11410667 PMC55748

[B45] Thoreson W. B. Dacey D. M. (2019). Diverse cell types, circuits, and mechanisms for color vision in the vertebrate retina. *Physiol. Rev.* 99 1527–1573. 10.1152/physrev.00027.2018 31140374 PMC6689740

[B46] Tohya S. Mochizuki A. Iwasa Y. (2003). Difference in the retinal cone mosaic pattern between zebrafish and medaka: cell-rearrangement model. *J. Theor. Biol.* 221 289–300. 10.1006/jtbi.2003.3192 12628235

[B47] Tsujimura T. Chinen A. Kawamura S. (2007). Identification of a locus control region for quadruplicated green-sensitive opsin genes in zebrafish. *Proc. Natl. Acad. Sci. U.S.A.* 104 12813–12818. 10.1073/pnas.0704061104 17646658 PMC1937549

[B48] Utagawa U. Higashi S. Kamei Y. Fukamachi S. (2016). Characterization of assortative mating in medaka: mate discrimination cues and factors that bias sexual preference. *Horm. Behav.* 84 9–17. 10.1016/j.yhbeh.2016.05.022 27260680

[B49] Valen R. Karlsen R. Helvik J. V. (2018). Environmental, population and life-stage plasticity in the visual system of Atlantic cod. *J. Exp. Biol.* 221:jeb165191. 10.1242/jeb.165191 29146770

[B50] Viets K. Eldred K. C. Johnston R. J. (2016). Mechanisms of photoreceptor patterning in vertebrates and invertebrates. *Trends Genet.* 32 638–659. 10.1016/j.tig.2016.07.004 27615122 PMC5035628

[B51] Ward W. W. Cormier M. J. (1979). Energy transfer protein in coelenterate bioluminescence. *J. Biol. Chem.* 254 781–788. 33175

[B52] Watanabe M. Furuya M. Miyoshi Y. Inoue Y. Iwahashi I. Matsumoto K. (1982). Design and performance of the okazaki large spectrograph for photobiological research. *Photochem. Photobiol.* 36 491–498. 10.1111/j.1751-1097.1982.tb04407.x

[B53] Yamanaka O. Takeuchi R. (2018). UMATracker: an intuitive image-based tracking platform. *J. Exp. Biol.* 221:jeb182469. 10.1242/jeb.182469 29954834

[B54] Young T. (1802). The bakerian lecture: on the theory of light and colours. *Philos. Trans. R. Soc. London* 92 12–48. 10.1098/rstl.1802.0004

[B55] Zimmermann M. J. Y. Nevala N. E. Yoshimatsu T. Osorio D. Nilsson D. E. Berens P. (2018). Zebrafish differentially process color across visual space to match natural scenes. *Curr. Biol.* 28 2018.e5–2032.e5. 10.1016/j.cub.2018.04.075 29937350

